# Cognitive confusion: A cross-sectional study on the cognitive differences between traditional tobacco and e-cigarettes among college students in Zhejiang Province, China

**DOI:** 10.18332/tid/215946

**Published:** 2026-02-19

**Authors:** Yuhuan Sun, Yang Yi, Geyao Huang, Shihao Jiang, Runze Chen, Dahui Wang, Falin Zhao

**Affiliations:** 1 School of Public Health and Nursing, Hangzhou Normal University, Hangzhou, China; 2 School of Public Administration, Hangzhou Normal University, Hangzhou, China; 3 Engineering Research Center of Mobile Health Management System, Ministry of Education, Hangzhou Normal University, Hangzhou, China

**Keywords:** traditional tobacco e-cigarettes, cognitive confusion, cross-sectional survey, college students

## Abstract

**INTRODUCTION:**

This study aimed to explore the differences in knowledge and cognitive perceptions of traditional tobacco and e-cigarettes among college students in Zhejiang Province, China, and to provide evidence-based insights for future prevention and control strategies.

**METHODS:**

A cross-sectional survey was conducted in September 2020 among students from nine undergraduate institutions in Zhejiang Province, selected through a combination of typical and convenience sampling. An anonymous online questionnaire was used to collect data on tobacco-related knowledge, usage behavior, and cognitive perceptions. Statistical analyses included chi-squared tests, rank-sum tests, logistic regression, McNemar's tests, and Cohen's kappa (κ) to assess differences and consistency in knowledge regarding traditional tobacco and e-cigarettes.

**RESULTS:**

A total of 728 valid responses were obtained. Among the respondents, 9.20% were current smokers and 6.73% were current e-cigarette users, of whom 1.37 % used e-cigarettes exclusively. Only 42.72% of respondents showed high knowledge of e-cigarettes, significantly lower than the 80.36% for traditional tobacco (χ^2^=128.410, p<0.001). Consistency in knowledge and risk perception between the two product types was also poor (κ<0.6). Among college students, 75.19% learned about e-cigarettes through the internet, and only 20.37% of those who had never used e-cigarettes correctly identified e-cigarette packaging.

**CONCLUSIONS:**

College students demonstrated significantly lower awareness of e-cigarettes compared to traditional tobacco, with notable cognitive inconsistency regarding health risks. The prevalence of misinformation, especially from online sources, underscores the urgent need for targeted education and control measures to address cognitive confusion and improve awareness of e-cigarette risks.

## INTRODUCTION

Electronic nicotine delivery systems (ENDS) or e-cigarettes, comprise vaporization devices and heat-not-burn tobacco products. These battery-powered apparatuses generate nicotine-containing aerosols through atomization processes without requiring the combustion of liquid solutions or tobacco sticks^1^. Although initially conceptualized as smoking cessation interventions to assist tobacco users in quitting or reducing cigarette consumption^2^, existing research indicates that the chemical constituents produced during ENDS operation may exert measurable physiological impacts.

The harm of e-cigarettes to adolescents and young adults is particularly concerning. In 2021, the World Health Organization (WHO) reiterated that e-cigarettes are not a means of quitting smoking, and they are harmful to public health and must be subject to stricter regulation, including a ban on sales to minors^3^. The irritants and carcinogens in e-cigarettes not only damage the digestive, cardiovascular, and respiratory systems^4,5^, but may also increase the risk of cancer^6^. Research consistently shows that e-cigarette vapor, once inhaled, can disrupt sleep^7^, blunt cognition, erode academic performance, and fuel anxiety and other negative emotions^5^. Emerging data also link vaping to an elevated risk of seizures in youth and young adults^8^. Beyond these physical harms, e-cigarette use is associated with depression^9^. Moreover, e-cigarettes that are modifiable can potentially be spiked with cannabis extracts such as tetrahydrocannabinol (THC) and cannabidiol (CBD)^10^. However, manufacturers often promote them as an improved version of cigarettes, emphasizing their fashionable and technological attributes^11^.

The use of e-cigarettes is highly likely to increase the future exposure of college students to traditional tobacco. Researchers argue that e-cigarettes serve as a gateway to traditional tobacco use^12^. Non-smokers who use e-cigarettes may transition to traditional tobacco, making e-cigarette use a strong risk factor for smoking among adolescents and young adults^13^. Additionally, a meta-analysis has shown that young adults (aged 14–30 years) who did not smoke but used e-cigarettes were 30% more likely to start smoking in the future, even after controlling for factors that could lead to smoking^14^.

Adolescents and young adults should be the primary focus of e-cigarette prevention efforts, as their use is increasing across multiple countries. The National College Health Assessment (NCHA) has noted that e-cigarette use in the past 30 days jumped from 4.9% in 2015 to 12.6% in 2019 among college students in the United States^15^. An Australian study on people aged 15–30 years shows that 14% of them are currently e-cigarette users, and 33% have tried or used e-cigarettes in the past^16^. Survey results consistently show that the prevalence of e-cigarette use among adolescents and young adults is higher than traditional tobacco use. A US adult study revealed that among individuals aged 18–24 years, the cigarette use rate was 10.4%, while the e-cigarette use rate reached as high as 32.9%. In China, although the smoking rate is lower among college students (7.8% in 2021), 10.1% of them have tried e-cigarettes^17^. A study by Zou et al.^18^ among Chinese college students found that 16.5% of students had tried e-cigarettes, 6.32% had used e-cigarettes in the past month, and 8.0% had the intention to use e-cigarettes. In Europe, the rate of e-cigarette use among those aged ≥15 years was 14.6% in 2017, with 25% of those aged 15–24 having tried them^18^.

Current research on young adults’ perceptions of e-cigarettes primarily has focused on the current state and the factors related to these perceptions, as well as the relationship between e-cigarette awareness and e-cigarette use. The results show that despite the confirmed negative health effects of e-cigarettes, young people still have some common misconceptions^19^. Both young adults who use traditional tobacco and those who use e-cigarettes believe that e-cigarettes are a healthier alternative to traditional tobacco. They think that the nicotine in e-cigarettes is less addictive and that e-cigarettes contain fewer chemicals, making them safer for those around them^20^.

In previous studies, traditional tobacco is often used as a reference for comparison with e-cigarettes, such as the statement ‘e-cigarettes are safer than traditional tobacco’. This type of questioning may introduce bias, making the survey results ‘favorable’ to e-cigarettes. This study employs an independent questioning method to compare college students’ awareness of traditional tobacco versus e-cigarettes and examines their cognitive perceptions through differential comparison and consistency analysis. This will provide a reference for e-cigarette prevention and control among adolescents, especially in terms of targeted strategies for tobacco control propaganda aimed at college students, to reduce current and future tobacco exposure among college students.

## METHODS

### Study design

We have carried out a cross-sectional study whereby a self-administered questionnaire was employed as an instrument for data collection to gather information pertaining to demographic and knowledge regarding traditional tobacco and e-cigarettes among college students in Zhejiang Province. The survey was completed in September 2020. The questionnaires were anonymous and self-administered by the respondents, who all provided informed consent. This study was approved by the Ethics Committee of Zhejiang Province.

### Setting and population

A combination of purposive sampling and convenience sampling was employed to select nine universities in Zhejiang Province, including one 985 university (a key university designated by the Ministry of Education, with only one in Zhejiang Province and eight ordinary undergraduate institutions. Among the eight ordinary universities, four are located in the provincial capital, Hangzhou, and the other four are in other cities in Zhejiang Province. In each university, electronic questionnaires were distributed to typical majors for an online survey. It was planned to distribute no fewer than 50 questionnaires in each university, with an appropriate increase in the number of questionnaires for universities with more majors and larger scales.

### Questionnaire survey

*Questionnaire*

This survey was conducted in the form of an online questionnaire, distributed via the WJX online survey platform. The questionnaire was designed based on the standardized recommendations for smoking surveys from the WHO^21^, and tailored to meet the specific objectives of this study. The content of the questionnaire includes the following sections: 1) General information – basic demographic data, including gender, grade, school type, ethnicity, major, monthly cost of living, and household registration; and 2) Knowledge of traditional tobacco and e-cigarettes – a total of 13 items were designed to assess participants’ knowledge in this area. Each correct answer was assigned 1 point, and the total score was calculated.

*Sample size*

Based on Kendall’s rough estimation method for sample size determination, the sample size was set to be 10 to 20 times the number of survey indicators. Given that the questionnaire contains 35 items, the initial sample size was determined to be at least 350 participants (10 times the number of items). Additionally, considering potential inefficiencies in questionnaire completion and inherent errors in the sampling method, the sample size was doubled. Therefore, the final number of participants required for the survey was noted to be at least 700.

### Quality control

Prior to the formal survey, a pilot study was conducted. Based on the results of the pilot study, the wording of some questions was refined to minimize ambiguity. For the online survey, each device was restricted to one response. The survey was limited to respondents within Zhejiang Province and required to be completed anonymously within a specified timeframe. In addition, quality control questions were embedded in the questionnaire to assess the attentiveness of the respondents. Any questionnaires with missing data in the sections on knowledge towards traditional tobacco and e-cigarettes were excluded from the analysis.

### Statistical analysis

The online questionnaire data were exported into Excel format and analyzed using SPSS 26.0^22^ statistical analysis software. Categorical data were described using frequencies and percentages. The rank-sum test was used to compare the total knowledge score between traditional tobacco and e-cigarettes. Factors associated with knowledge level were analyzed using univariate chi-squared tests followed by logistic regression. Unlike the conventional practice of using 60% of the total score as the cutoff, higher thresholds are generally adopted in health knowledge studies. Consistent with the method of Aghar et al.^23^, we dichotomized the total knowledge score at the 75th percentile (high ≥10 points; low <10 points) and employed this binary variable as the outcome measure in logistic regression models^23^. Multivariable logistic regression with backward stepwise selection (entry α <0.1, removal α >0.05) was used to identify factors associated with knowledge level. Variables remaining in the final model were deemed statistically significant. McNemar’s test was used to compare the accuracy rates of each knowledge question between tobacco and e-cigarettes. Accuracy rate was defined as the proportion of participants who answered each knowledge question correctly, expressed as: (number of correct responses/total number of responses) × 100%. Cohen’s kappa was employed to analyze the cognitive agreement among college students regarding traditional tobacco and e-cigarettes. All hypothesis tests were two-sided, with a significance level of α=0.05.

## RESULTS

### Basic information and tobacco prevalence

A total of 763 questionnaires were collected online in this survey, of which 728 were valid, resulting in an effective response rate of 95.4%. Among the college students who participated in the survey, 429 (58.93%) were female, 705 (96.84%) were Han Chinese, and 401(55.08%) were registered in rural areas. Students in their third year or above were classified as the senior group, comprising 506 individuals (69.51%). A total of 635 participants (87.23%) were from ordinary universities, while 93 students (12.77%) were from ‘985’ universities. There were 101 students majoring in medicine, representing 13.87%. Among the surveyed students, 368 (50.55%) had at least one parent who smoked, and 293 (40.25%) had at least one friend who smoked. Currently, 67 students (9.20%) were smokers, and 49 (6.73%) were using e-cigarettes, of whom 10 (1.37%) used only e-cigarettes. A total of 81 students (11.1%) had ever used e-cigarettes (Table 1).

**Table 1:** Univariate analysis of factors influencing the level of knowledge of traditional tobacco and e-cigarettes among college students, Zhejiang, China (N=728)

*Characteristics*	*Categories*	*Total n (%)*	*Traditional tobacco* *high knowledge level*		*E-cigarette high* *knowledge level*	
			*n (%)*	p*	*n (%)*	p*
**Gender**	Male	299 (41.07)	227 (75.92)	0.012	124 (41.47)	0.570
	Female	429 (58.93)	358 (83.45)		187 (43.59)	
**Ethnicity**	Han Chinese	705 (96.84)	575 (81.56)	<0.001	302 (42.84)	0.724
	Minority	23 (3.16)	10 (43.48)		9 (39.13)	
**Residence**	Urban	327 (44.92)	263 (80.43)	0.965	147 (44.95)	0.271
	Rural	401 (55.08)	322 (80.30)		164 (40.90)	
**School type**	985 University	93 (12.77)	81 (87.10)	0.080	41 (44.09)	0.776
	Undergraduate	635 (87.23)	504 (79.37)		270 (45.52)	
**Grade**	Lower	222 (30.49)	176 (79.28)	0.628	95 (42.79)	0.979
	Senior	506 (69.51)	409 (80.83)		216 (42.69)	
**Major**	Medicine	101 (13.87)	93 (92.08)	<0.001	44 (43.56)	0.853
	Other	627 (86.13)	492 (78.47)		267 (42.58)	
**Monthly cost of living (RMB)**	0–2000	552 (75.82)	439 (79.53)	0.319	226 (40.94)	0.086
	>2000	176 (24.18)	146 (82.95)		85 (48.30)	
**Smoking of parents**	Yes	368 (50.55)	298 (80.98)	0.670	151 (41.03)	0.352
	No	360 (49.45)	287 (79.72)		160 (44.44)	
**Smoking with friends**	Yes	293 (40.25)	237 (80.89)	0.768	139 (47.44)	0.035
	No	435 (59.75)	348 (80.00)		172 (39.54)	
**Traditional tobacco use**	Yes	67 (9.20)	42 (62.69)	<0.001	22 (32.84)	0.086
	No	661 (90.80)	543 (82.15)		289 (43.72)	
**E-cigarette use**	Yes	49 (6.73)	23 (46.94)	<0.001	13 (26.53)	0.018
	No	679 (93.27)	562 (82.77)		298 (43.89)	

* Chi-squared tests. RMB: 1000 Chinese Renminbi about US$140.

### Analysis of factors related to knowledge levels of traditional tobacco and e-cigarettes among college students

Univariate analysis revealed that gender, ethnicity, major, and traditional tobacco use were not significantly related to college students’ knowledge level of e-cigarettes, but were related to their knowledge level of traditional tobacco (Table 1). Specifically, among male students, the proportion with a high level of knowledge about traditional tobacco was 75.92%, lower than that of female students (83.45%). Han Chinese students had a significantly higher proportion of high knowledge level about traditional tobacco (81.56%) compared to non-Han students (43.48%). Students majoring in medicine exhibited a higher proportion of high knowledge level about traditional tobacco (92.08%) than others (78.47%). Additionally, non-smokers of traditional tobacco had a higher proportion of high knowledge level about traditional tobacco (82.15%) than smokers (62.69%). E-cigarette use significantly affected the knowledge levels of both traditional tobacco and e-cigarettes among college students. Notably, non-users of e-cigarettes had a higher proportion of high knowledge levels for both traditional tobacco and e-cigarettes compared to e-cigarette users (82.77% vs 46.94% for e-cigarettes; 43.89% vs 26.53% for traditional tobacco). All differences were statistically significant (p<0.05).

A multivariable binary logistic regression analysis was conducted with the level of traditional tobacco knowledge as the dependent variable, and gender, ethnicity, major (medicine vs other), smoking status, and e-cigarette usage as independent variables. The results revealed that medical students (AOR=2.90; 95% CI: 1.36–6.18), Han ethnicity students (AOR=4.93; 95% CI: 1.96–12.44), and non-e-cigarette users (AOR=3.94; 95% CI: 2.10–7.41) had a higher level of traditional tobacco knowledge. A multivariable logistic regression analysis was also performed with e-cigarette knowledge as the dependent variable and e-cigarette usage, smoking of friends as the independent variable, which showed that the students who have smoking of friends (AOR=1.51; 95% CI: 1.11–2.05) and non-e-cigarette users (AOR=2.54; 95% CI: 1.31–4.95) had a higher level of e-cigarette tobacco knowledge (Table 2).

**Table 2:** Logistic regression analysis of factors influencing the level of knowledge of traditional tobacco and e-cigarettes among college students, Zhejiang, China (N=728)

*Product*	*Variables*		*AOR (95% CI)*
**Traditional tobacco**	Ethnicity	Han Chinese vs Minority (ref.)	4.93 (1.96–12.44) ***
	Major	Medicine vs Other (ref.)	2.90 (1.36–6.18) **
	E-cigarette smoking	No vs Yes (ref.)	3.94 (2.10–7.41) ***
**E-cigarettes**	Smoking of friends	No (ref.) vs Yes	1.51 (1.11–2.05) **
	E-cigarette smoking	No vs Yes (ref.)	2.54 (1.31–4.95) **

AOR: adjusted odds ratio.

* p<0.05.

** p<0.01.

*** p<0.001.

### Comparison of college students’ knowledge scores of traditional tobacco and e-cigarettes

*Analysis of the differences in knowledge scores between traditional tobacco and e-cigarettes*

**Figure 1:**
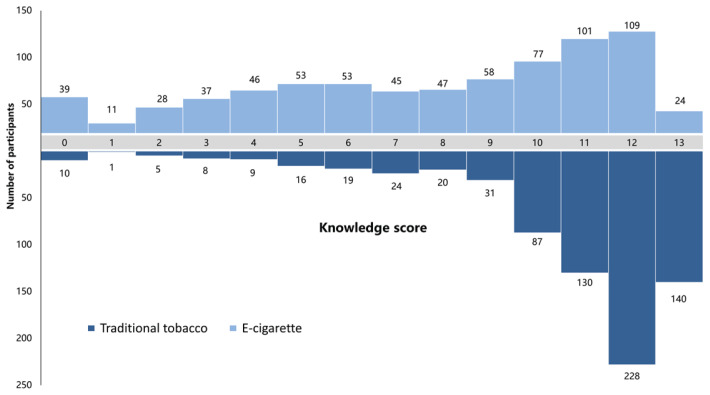
Distribution of college students’ traditional tobacco and e-cigarette knowledge scores, Zhejiang, China (N=728)

The results of this survey indicate that among college students, 585 individuals (80.36%) had a higher level of knowledge about traditional tobacco (total score ≥10 points), while only 311 individuals (42.72%) demonstrated a higher level of knowledge about e-cigarettes. In comparison, fewer students had a higher level of knowledge about e-cigarettes (χ^2^=128.410, p<0.001). Specifically, 140 participants (19.23%) correctly answered all 13 items related to traditional tobacco knowledge, whereas only 24 participants (3.30%) correctly answered all 13 items related to e-cigarette knowledge. The level of knowledge about traditional tobacco was significantly higher than that of e-cigarettes (Z= -19.741, p<0.001) (Figure 1).

Differences in the knowledge scores of 728 college students about traditional tobacco and e-cigarettes were compared using a paired sample rank-sum test, and the differences were statistically significant (Z= -19.741, p<0.001).

Using the McNemar’s test, we found that there were statistically significant (p<0.001) differences in the awareness of traditional tobacco and e-cigarettes for all 13 items (Table 3). Among the 13 items related to e-cigarettes, the item with the highest error rate was ‘E-cigarettes can be purchased from e-commerce platforms’, with an error rate of 83.14%. The item with the lowest error rate was ‘E-cigarettes are suitable for pregnant women’ with an error rate of only 16.62%. For the 13 items related to traditional tobacco, 57.01% of the respondents incorrectly believed that ‘Traditional tobacco can be purchased from e-commerce platforms’. The question with the lowest error rate was ‘Traditional tobacco is harmless’, with an error rate of only 7.97%.

**Table 3:** College students’ responses accuracy rate on specific knowledge about traditional tobacco and e-cigarettes, Zhejiang, China (N=728)

*No.*	*Items*	*Traditional tobacco* *n (%)*	*E-cigarettes* *n (%)*	p*
1	It’s harmless	670 (92.03)	479 (65.80)	<0.001
2	It does not contribute to secondhand smoking	658 (90.38)	422 (57.97)	<0.001
3	It is suitable for pregnant women	658 (90.38)	607 (83.38)	<0.001
4	It is not addictive	654 (89.84)	530 (72.80)	<0.001
5	It impairs lung function	649 (89.15)	476 (65.38)	<0.001
6	It is associated with lung cancer	640 (87.91)	412 (56.59)	<0.001
7	It can be sold to minors	632 (86.81)	584 (80.22)	<0.001
8	It is suitable for teenagers	630 (86.54)	563 (77.34)	<0.001
9	It can affect fetal development	627 (86.13)	504 (69.23)	<0.001
10	Most of it contains nicotine	618 (84.89)	272 (37.36)	<0.001
11	It is associated with heart disease	528 (72.53)	318 (43.68)	<0.001
12	It is associated with bladder cancer	451 (61.95)	369 (50.69)	<0.001
13	It can be purchased from e-commerce platforms	313 (42.99)	123 (16.86)	<0.001

Accuracy rate was defined as the proportion of participants who answered each knowledge question correctly, expressed as: (number of correct responses/total number of responses) × 100%.

* Paired chi-square test (McNemar’s test).

*Consistency analysis of college students’ responses to knowledge about traditional tobacco and e-cigarettes*

A scatter plot was constructed using the kappa coefficient – representing the cognitive consistency between perceptions of traditional tobacco and e-cigarettes as the x-axis and the accuracy rate of responses as the y-axis. Reference lines were set at a kappa value of 0.5 and an accuracy rate of 75%, dividing the plot into four quadrants: Quadrant I – high accuracy and high cognitive consistency; Quadrant II – high accuracy but low cognitive consistency; Quadrant III – low accuracy and low cognitive consistency; and Quadrant IV – low accuracy but high cognitive consistency

**Figure 2:**
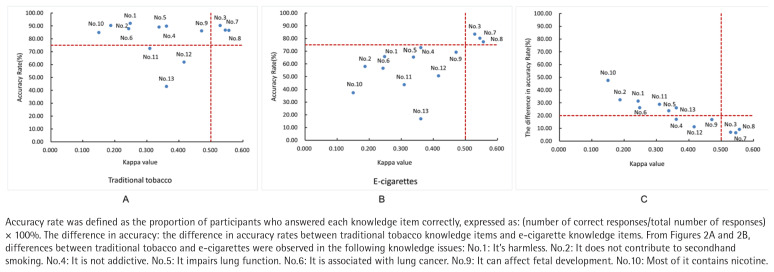
Scatter plot of the awareness accuracy rates versus corresponding kappa values for traditional tobacco and e-cigarettes, Zhejiang, China

As shown in Figures 2A and 2B, items 3, 7, and 8 fall into Quadrant I. The items ‘Can be used by pregnant women’, ‘Can be sold to minors’, and ‘Can be used by adolescents’ are primarily related to basic regulatory knowledge, and show both high accuracy and high consistency among respondents. In contrast, items 11, 12, and 13 are consistently located in Quadrant III for both traditional tobacco and e-cigarettes. The items ‘Associated with heart disease’, ‘Associated with bladder cancer’, and ‘Available through e-commerce platforms’ exhibit both low accuracy and low cognitive consistency, indicating widespread gaps in knowledge among participants. Among the remaining seven items, all responses regarding traditional tobacco are situated in Quadrant II, while those concerning e-cigarettes tend to cluster in Quadrant III. These seven items reflect the main differences in college students’ perceptions of traditional tobacco and e-cigarettes. Furthermore, a clear negative correlation is observed between the difference in accuracy rates and the kappa coefficient (Figure 2C), suggesting that lower cognitive accuracy is strongly associated with greater inconsistency in perceptions between traditional tobacco and e-cigarettes.

### Channels to access information about e-cigarettes and identification of their packaging

Regarding the channels to access information about e-cigarettes, 75.19% of students learned about them through the internet, and 48.88% were informed by friends or family members; 31.54% of students knew e-cigarettes through television (Supplementary file Figure S1A). Notably, among college students who had never used e-cigarettes, only 20.37% correctly identified e-cigarette packaging. Misidentifications were common, with 2.47% confusing a USB drive, 8.18% a lighter, and 68.98% a metal pen for an e-cigarette (Supplementary file Figure S1B).

## DISCUSSION

This study employed an independent questioning approach, which allows for a more direct comparison of college students’ knowledge of traditional tobacco and e-cigarettes. This method contrasts with previous studies that used traditional tobacco as a reference for comparison with e-cigarettes, thereby minimizing the directional bias that could arise from such an approach. Our aim was to reduce potential biases, ensuring that the findings are more objective. Furthermore, we employ differential comparison and consistency analysis to examine college students’ cognitive understanding of traditional tobacco versus e-cigarettes. This research design provides more reliable data to support efforts in preventing and controlling e-cigarette use among college students.

The survey revealed that both e-cigarette and traditional tobacco use among college students in Zhejiang Province exceed national averages, with 6.73% currently using e-cigarettes and 11.1% having ever used them – rates higher than the national values of 2.5% and 10.1%, respectively, reported in the 2021 China College Student Tobacco Control Study^17^. Similarly, the current smoking rate for traditional tobacco (9.20%) also surpasses the national average of 7.8% among college students^17^. These elevated usage rates may reflect the unique socioeconomic and cultural context of Zhejiang, an economically developed region in East China, where students may be more influenced by fashion trends and novelty. Notably, while over 80% of students demonstrated a high level of knowledge about traditional tobacco, only 42.72% did so regarding e-cigarettes. Across all 13 knowledge-related items, students scored consistently higher for traditional tobacco than for e-cigarettes. Additionally, nearly 80% of non-users were unable to correctly identify e-cigarette products based on appearance, suggesting a widespread lack of awareness about e-cigarette forms and features. These findings highlight a significant knowledge gap regarding e-cigarettes despite their increasing prevalence among college students.

The quadrant-based consistency analysis further underscores disparities in perception. Items positioned in Quadrant I – indicating both high accuracy and high cognitive consistency – primarily pertain to legal and regulatory issues, such as use during pregnancy, sale to minors, and adolescent use (items 3, 7, and 8). The consistent understanding of these topics across both product types suggests that public health initiatives and regulatory education have been relatively successful in these domains. In contrast, items 11, 12, and 13, which focus on health risks like heart disease and bladder cancer, as well as the legality of online sales, fall into Quadrant III for both products, reflecting substantial knowledge gaps that warrant immediate attention in health education efforts. The remaining items show a clear asymmetry: while students demonstrate relatively accurate knowledge of traditional tobacco (Quadrant II), their understanding of e-cigarettes is notably weaker (Quadrant III), with a low level of cognitive consistency. These items represent the primary source of confusion in college students’ perceptions of traditional tobacco versus e-cigarettes. This discrepancy likely explains why students perceive e-cigarettes as distinct from traditional tobacco and highlights key areas requiring clarification and reinforcement in e-cigarette health education. Misconceptions about e-cigarette content and health impacts such as their nicotine composition, secondhand smoke potential, addictiveness, and links to diseases, are widespread and likely exacerbated by misleading marketing that downplays potential harms. The strong negative correlation between the accuracy gap and the kappa coefficient further supports the conclusion that limited knowledge contributes directly to cognitive inconsistencies.

Overall, college students’ knowledge of e-cigarettes remains significantly deficient compared to their understanding of traditional tobacco. They lack awareness of the harmful effects and do not fully recognize that e-cigarettes are a form of tobacco. Manufacturers downplay the risks in their advertising, using tactics like celebrity endorsements and cartoon imagery to present e-cigarettes as fashionable and trendy, which appeals to students’ curiosity and prompts experimentation^24^. Studies from the US^25^, South Korea^26^, and Finland^27^ have shown that curiosity is a key factor driving adolescents to try e-cigarettes. This study supports the effectiveness of these marketing strategies. Furthermore, the primary channels to access information about e-cigarettes for students are the internet, where e-cigarettes are often marketed as smoking cessation aids and a healthier, more affordable alternative to tobacco^28^. Driven by profit, manufacturers have marketed e-cigarettes as trendy items for young people, contributing to rising usage rates^29^.

The insufficient public awareness campaigns on the dangers of e-cigarettes by society and educational institutions have contributed to many college students’ overestimation of e-cigarettes as harmless or as a tool for smoking cessation^24^. As Yao et al.^30^ found in a study of 18 websites of 12 e-cigarette manufacturers in China, the most frequently mentioned health-related benefits in e-cigarette advertisements (89%) were claims of no secondhand smoke exposure (78%) and effectiveness as a smoking cessation aid (67%). The advertisements also featured a variety of flavors, celebrity endorsements, and e-cigarettes marketed specifically for women^30^. The results of this study similarly show that college students hold misconceptions about the harms of e-cigarettes and secondhand smoke exposure. These marketing strategies and sales tactics contribute to the misperceptions of e-cigarettes among college students, leading them to use e-cigarettes incorrectly.

Currently, about 35 countries/regions have banned ENDS. In countries where e-cigarettes are sold, the WHO recommends stringent regulations to limit their appeal and reduce harm, such as banning flavors, restricting nicotine levels, and imposing taxes^31^. The ‘Measures for the Administration of E-Cigarettes’ issued by the State Tobacco Monopoly Administration require public education on e-cigarette risks, discouragement of adolescent use, and the ban of use in primary and secondary schools^32^. E-cigarette packaging must comply with requirements for labeling, health warnings, and packaging, including messages like ‘E-cigarettes are harmful to health’, ‘Quitting is beneficial to health’, and ‘Discourage adolescent use’. Additionally, regulations prohibit misleading claims like ‘health benefits’, ‘low risk’, and terms that could entice minors, such as ‘light’ or ‘mild’ on e-cigarette packaging and labels^33^. Vassey et al.^34^ noted that changes to nicotine warning labels could reduce their effectiveness in deterring e-cigarette use.

Additionally, Margolis et al.^35^ have also demonstrated in their study that, among students who have never smoked, those who perceive e-cigarettes as highly harmful exhibit a lower acceptance of e-cigarettes compared to those who perceive them as low-risk. This finding aligns with the results of the present study, where the low level of awareness regarding both traditional tobacco and e-cigarettes is identified as a risk factor for tobacco use. Among the college students who do not use e-cigarettes, there is often a more comprehensive understanding of e-cigarettes, suggesting that a lack of adequate information may contribute to improper use of e-cigarettes. Furthermore, college students who use e-cigarettes generally have a lower level of health knowledge, and this interaction may facilitate a harmful cycle of e-cigarette use. The low level of awareness is likely a significant factor driving the increasing usage rate.

Therefore, if adolescents and young adults struggle to establish a correct understanding of the long-term health risks, once they begin using tobacco products, they are more likely to become lifelong users^36^. Moreover, using e-cigarettes with higher nicotine concentrations may increase the frequency and intensity of smoking. Consequently, it is imperative to enhance the education of college students about the harms of e-cigarettes and urgently develop a systematic, accurate knowledge framework about e-cigarettes, similar to that of traditional tobacco.

In summary, e-cigarette manufacturers may have employed promotional strategies that ‘obfuscate the tobacco nature of e-cigarettes’ and ‘promote e-cigarette products as fashionable’, and accelerated their dissemination through the Internet. These strategies are likely associated with the cognitive biases of college students regarding e-cigarettes. To better control e-cigarette usage, it is essential to clearly define the tobacco nature of e-cigarettes in health education and public communication, emphasizing that e-cigarette users are also smokers. Relevant regulatory policies should be implemented to prohibit or restrict the use of biased language in e-cigarette advertisements, and clear labeling such as ‘Using e-cigarettes is harmful to health’ and ‘Please do not use e-cigarettes in non-smoking areas’ should be enforced to strengthen the dissemination of accurate information. This approach will help convey clear and accurate knowledge about e-cigarettes to the public, particularly adolescents, dismantle the ‘fashionable’ label associated with e-cigarettes, and improve overall awareness of e-cigarettes.

### Limitations

This study has several limitations. First, although the study expanded its scope by using an online survey method, the lack of probability sampling may limit the representativeness of the results. Second, the survey was conducted only in Zhejiang Province, a region in southeastern coastal China with a relatively high level of economic development. As a result, the findings may not be applicable to provinces with different economic profiles. Third, the reliance on a self-administered questionnaire to assess knowledge and behaviors may introduce misclassification bias. Fourth, this study is cross-sectional, and causal inferences cannot be made. Fifth, despite adjustment for key sociodemographic covariates, residual confounding from unmeasured factors (e.g. mental-health status) cannot be ruled out. The above limitations may affect the extrapolation of the results obtained in this study and need to be addressed in future studies.

## CONCLUSIONS

The prevalence of traditional tobacco and e-cigarette use among our sample of college students in Zhejiang Province was found to be higher than the national average for Chinese college students in 2021 (6.73% vs 2.5% for e-cigarettes, and 9.20% vs 7.8% for traditional tobacco). Knowledge levels regarding e-cigarettes were observed to be lower than those for traditional tobacco. Furthermore, the consistency of college students’ awareness of the risks associated with both traditional tobacco and e-cigarettes was found to be low, with widespread confusion about the nature and health impacts of e-cigarettes. These findings highlight the need for targeted tobacco control efforts among college students, emphasizing the importance of increasing awareness about the risks of e-cigarettes, providing accurate information, correcting misconceptions, especially clarifying that both traditional tobacco and e-cigarettes are fundamentally tobacco products.

## Supplementary Material



## Data Availability

The data supporting this research are available from the authors on reasonable request.
